# Cuproptosis-Associated lncRNA Establishes New Prognostic Profile and Predicts Immunotherapy Response in Clear Cell Renal Cell Carcinoma

**DOI:** 10.3389/fgene.2022.938259

**Published:** 2022-07-15

**Authors:** Shengxian Xu, Dongze Liu, Taihao Chang, Xiaodong Wen, Shenfei Ma, Guangyu Sun, Longbin Wang, Shuaiqi Chen, Yong Xu, Hongtuan Zhang

**Affiliations:** ^1^ Department of Urology, National Key Specialty of Urology Second Hospital of Tianjin Medical University Tianjin Key Institute of Urology Tianjin Medical University, Tianjin, China; ^2^ Department of Family Planning, The Second Hospital of Tianjin Medical University, Tianjin, China

**Keywords:** cuproptosis, lncRNA, ccRCC, prognostic model, bioinformatics

## Abstract

**Background:** Clear cell renal cell carcinoma (ccRCC) accounts for 80% of all kidney cancers and has a poor prognosis. Recent studies have shown that copper-dependent, regulated cell death differs from previously known death mechanisms (apoptosis, ferroptosis, and necroptosis) and is dependent on mitochondrial respiration (Tsvetkov et al., Science, 2022, 375 (6586), 1254–1261). Studies also suggested that targeting cuproptosis may be a novel therapeutic strategy for cancer therapy. In ccRCC, both cuproptosis and lncRNA were critical, but the mechanisms were not fully understood. The aim of our study was to construct a prognostic profile based on cuproptosis-associated lncRNAs to predict the prognosis of ccRCC and to study the immune profile of clear cell renal cell carcinoma (ccRCC).

**Methods:** We downloaded the transcriptional profile and clinical information of ccRCC from The Cancer Genome Atlas (TCGA). Co-expression network analysis, Cox regression method, and least absolute shrinkage and selection operator (LASSO) method were used to identify cuproptosis-associated lncRNAs and to construct a risk prognostic model. In addition, the predictive performance of the model was validated and recognized by an integrated approach. We then also constructed a nomogram to predict the prognosis of ccRCC patients. Differences in biological function were investigated by GO, KEGG, and immunoassay. Immunotherapy response was measured using tumor mutational burden (TMB) and tumor immune dysfunction and rejection (TIDE) scores.

**Results:** We constructed a panel of 10 cuproptosis-associated lncRNAs (HHLA3, H1-10-AS1, PICSAR, LINC02027, SNHG15, SNHG8, LINC00471, EIF1B-AS1, LINC02154, and MINCR) to construct a prognostic prediction model. The Kaplan–Meier and ROC curves showed that the feature had acceptable predictive validity in the TCGA training, test, and complete groups. The cuproptosis-associated lncRNA model had higher diagnostic efficiency compared to other clinical features. The analysis of Immune cell infiltration and ssGSEA further confirmed that predictive features were significantly associated with the immune status of ccRCC patients. Notably, the superimposed effect of patients in the high-risk group and high TMB resulted in shorter survival. In addition, the higher TIDE scores in the high-risk group suggested a poorer outcome for immune checkpoint blockade response in these patients.

**Conclusion:** The ten cuproptosis-related risk profiles for lncRNA may help assess the prognosis and molecular profile of ccRCC patients and improve treatment options, which can be further applied in the clinic.

## Introduction

Renal cell carcinoma is a common genitourinary malignancy that causes nearly 170,000 deaths each year. The most common histologic type of renal cell carcinoma is clear cell renal cell carcinoma (ccRCC), which accounts for approximately 80% of cases (Delman, 2020). Due to the asymptomatic nature of renal clear cell carcinoma, metastases are usually already present at the time of diagnosis. Surgery is also difficult to remove renal cell carcinoma metastases, and recurrence is common after nephrectomy. Also, ccRCC differs from other urologic tumors in that it is insensitive to both radiotherapy and chemotherapy (Ljungberg et al., 2015). As a highly immunogenic tumor, ccRCC may benefit from immunotherapy. Although immunotherapy has indeed made considerable breakthroughs in ccRCC, treatment outcomes still vary from individual to individual (Motzer et al., 2019). Therefore, there is an urgent need to better understand the heterogeneity of ccRCC patients and establish an accurate and comprehensive risk model to stratify patients to design personalized treatment plans in terms of prognosis prediction and drug selection.

Long non-coding RNA (lncRNA) refers to RNAs that are longer than 200bp and do not have protein-coding functions, which play an important regulatory role in immune response processes, such as immune cell infiltration, antigen recognition, antigen exposure, and tumor clearance (Quinn and Chang, 2016).

LncRNAs play specific roles in carcinogenesis and metastasis by transcription and post-transcriptional modifications of genes (Du et al., 2020; Gao et al., 2020; Liu et al., 2021). Lv pointed out that lncRNAs were associated with tumor autophagy in ccRCC(9). At the same time, a number of studies have shown that lncRNAs can influence the expression of target genes by acting as competing RNAs (Liu and Lei, 2021; Shan et al., 2022a; Zhang et al., 2022). LncRNAs are also connected to drug resistance in tumors (Barik et al., 2021). However, studies on the role of cuproptosis-associated lncRNAs in ccRCC prognosis and tumor immunity (TIME) are still unclear.

Copper is an indispensable cofactor for all organisms to maintain life activities, as it plays an important role in biological processes such as mitochondrial respiration, antioxidant/detoxification, and iron uptake (Ruiz et al., 2021). However, it can become harmful if the concentration of copper in the body exceeds the threshold that can be maintained by homeostatic mechanisms. Recent studies have indicated that copper-regulated cell death occurs in a manner that is different from previously known death mechanisms (apoptosis, ferroptosis, and necroptosis) and that it is closely linked to mitochondrial respiration. Specifically, cuproptosis occurs through direct binding of copper to the lipidated components of the tricarboxylic acid (TCA) cycle. The combination of the two will lead to lipid-acylated protein aggregation and subsequent loss of iron-sulfur cluster proteins, further leading to proteotoxic stress and ultimately cell death (Tsvetkov et al., 2022). Several links have been observed between copper and cancer. Copper accumulation is closely associated with tumor cell development, angiogenesis, and metastasis (Lelièvre et al., 2020; Li, 2020; Ruiz et al., 2021; Ge et al., 2022). Currently, the mechanism of copper-mediated death regulation in tumors is unclear, and studies on the role of copper-death-associated lncRNAs in ccRCC are inconclusive. Therefore, our study aims to explore the role of cuproptosis-related lncRNAs in ccRCC using bioinformatics.

## Materials and Methods

### Data Collection

RNA sequencing data and clinical characterization data for ccRCC were obtained on 9 April 2022 by downloading from the TCGA database (https://portal.gdc.cancer.gov/repository), which included a dataset of 539 tumor samples and a dataset of 72 normal tissue samples (Liu et al., 2018). Using the Perl programming language (version Strawberry-Perl-5.30.0; https://www.perl.org), the RNA-seq data were extracted in the fragment per kilobase million (FPKM) format that has been normalized (Conesa et al., 2016). At the same time, the clinical data were preprocessed with Pearl to obtain the complete pathological information of the clinical samples.

### Screening and Differential Expression Analysis of Cuproptosis-Associated lncRNAs

Using the packages “limma,” “dplyr,” “ggalluvial,” and “ggplot2,” we plotted the Sankey relationship between cuproptosis genes and cuproptosis-associated lncRNAs (Ritchie et al., 2015). Our team was filtered using Pearson’s correlation analysis with the criteria of |Pearson R| > 0.4 and *p* < 0.001.

### Modeling and Validation of Prognostic Risk Assessment

The KIRC dataset from TCGA was randomly divided into a training risk set and a test risk set using the caret R package in a 1:1 ratio. The train set was utilized to construct cuproptosis-related lncRNA signatures, and the test set and the whole set were applied to validate the signature.

Univariate Cox regression analysis was applied to identify prognosis-associated lncRNAs among those cuproptosis-associated lncRNAs (*p* < 0.05), and forest plots were drawn. Also, we mapped these lncRNAs by the “limma,” “pheatmap,” “reshape2,″ and “ggpubr” packages. Then, by performing LASSO Cox regression algorithm analysis (using the penalty parameter estimated by 10-fold cross-validation) on the obtained prognostic lncRNAs, we determined the best group of prognostic lncRNAs and established the risk model. This approach minimizes overfitting in the modeling process. Finally, we developed a prognostic risk model based on optimal lncRNA using multivariate Cox regression and calculated the risk score for each patient with ccRCC according to the following equation:

risk score=∑i=1nCoef(i)×Expr(i).

Coef (i) and Expr (i) in the formula denote the regression coefficient of the multiple Cox regression analysis for each lncRNA and normalized expression level for each lncRNA, respectively. The median of the training set was used as a cut-off point to classify all samples containing KIRC as low- or high-risk subsets. Kaplan–Meier (KM) curves were adopted to explore whether there is a difference in the overall survival and progression-free survival of ccRCC patients between the high-risk and low-risk subsets in the training and testing sets using the “survival” R package. The chi-square test was utilized by us to evaluate the correlation between the model and the clinical characteristics. Based on survival, caret, glmnet, rms, survminer, and timeROC packages, we generated ROC curves and calculated the area under the curve (AUC) and applied the consistency index (C-index) together to measure the accuracy of the model.

### Nomogram and Calibration

Combining risk scores with various clinical pathological factors, the rms package was applied to create line graphs for 1-, 3-, and 5-years OS for ccRCC patients. The calibration curve based on the Hosmer–Lemeshow test was used to show the predictive power of the nomogram models developed.

### PCA, GO, and KEGG Analysis

The expression patterns of cuproptosis-related lncRNAs for ccRCC samples were classified using principal component analysis to visualize the spatial distribution of high- and low-risk samples. In addition, for the differential genes in the low- and high-risk groups, we used Gene Ontology (GO) analysis, which consisted of three components: biological process (BP), cellular component (CC), and molecular function (MF). Also, differentially expressed KEGG pathways in the two groups were analyzed using the Hs. eg.db, clusterProfiler, and enrichplot packages. *p* < 0.05 and FDR <0.05 were considered as significantly enriched biological processes and pathways.

### Tumor Immune Analysis

In order to explore the relationship between this model and immune infiltration status, our team calculated the immune infiltration profile of the TCGA-KIRC dataset using seven algorithms (XCELL, TIMER, QUANTISEQ, MCPCOUNTER, EPIC, CIBERSORT-ABS, and CIBERSORT) (Aran et al., 2017; Li et al., 2017; Racle et al., 2017; Chen et al., 2018; Dienstmann et al., 2019; Finotello et al., 2019; Li et al., 2020; Tamminga et al., 2020). Wilcoxon signed-rank test, limma, tidyverse, scales, ggplot2, and ggtext R packages were used to perform the analysis of the differences in the content of immune infiltrating cells in the different risk groups explored and the outcomes were shown in the bubble plots.

Then, based on the ESTIMATE algorithm, we explored the abundance of immune and stromal cells between different groups and calculated the StromalScore, ImmuneScore, and ESTIMATEScore (StromalScore + ImmuneScore) for each group (Chen et al., 2018). In addition, we investigated the differential expression of immune checkpoints in high- and low-risk populations and showed them in box plots. Subsequently, single-sample GSEA (ssGSEA) scoring of infiltrating immune cells and immune-related functions in ccRCC was performed by the “limma,” “GSVA,” and “GSEABase” packages and presented as a heat map.

### Tumor Mutation Burden and Tumor Immune Dysfunction and Exclusion Score

After downloading the somatic mutation data from the TCGA website, we applied the Pearl programming language to extract the mutation data. Then, we examined and integrated TCGA data using the “maftools” package and analyzed the differences in TMB and survival rates between the high-risk and low-risk groups. The tumor immune dysfunction and exclusion (TIDE) scoring file was retrieved from the TIDE website (http://tide.dfci.harvard.edu) (Jiang et al., 2018). We then assessed potential differences in immune checkpoint blockade (ICB) responses between the low- and high-risk groups using the “ggpubr” package. Finally, our team used the R package pRRophetic to predict the IC50 values of drugs available for the treatment of ccRCC in the high- and low-risk groups.

### Validation of the Expression Level of Screened Hub Cuproptosis-Associated lncRNAs in KIRC by qRT-PCR

Cancer and adjacent normal tissues were collected from six patients with renal clear cell carcinoma admitted to the Second Hospital of Tianjin Medical University. Each patient was informed and signed the consent form. The study was approved by the Institutional Review Board of the Second Hospital of Tianjin Medical University. All tissues were rapidly stored in liquid nitrogen after excision. After tissue grinding, total RNA was extracted from ccRCC tissue using TRIzol reagent (Invitrogen, China) according to the manufacturer’s protocol. Finally, we performed a quantitative reverse transcription-polymerase chain reaction (qRT-PCR) on cDNA using FastStart Universal SYBR Green Master (ROX, Roche; United States). GAPDH was used as a reference. The following primer sequences were used: GAPDH-F: GGA​AGG​TGA​AGG​TCG​GAG​TCA, GAPDH-R: GTC​ATT​GAT​GGC​AAC​AAT​ATA​TCC​ACT; SNHG15-F: TGG​CAG​ACC​TGT​ACT​CCG​TA, SNHG15-R: CCT​GGG​CTC​AGG​AAT​GGT​CA; LINC00471-F: TAT​CAC​CAA​GCA​GGA​GGG​GA, LINC00471-R: ATC​GGG​AAC​CCC​CTA​CAG​AA.

## Results

### Prognosis-Related lncRNAs With Coexpression of Cuproptosis

**FIGURE 1:**
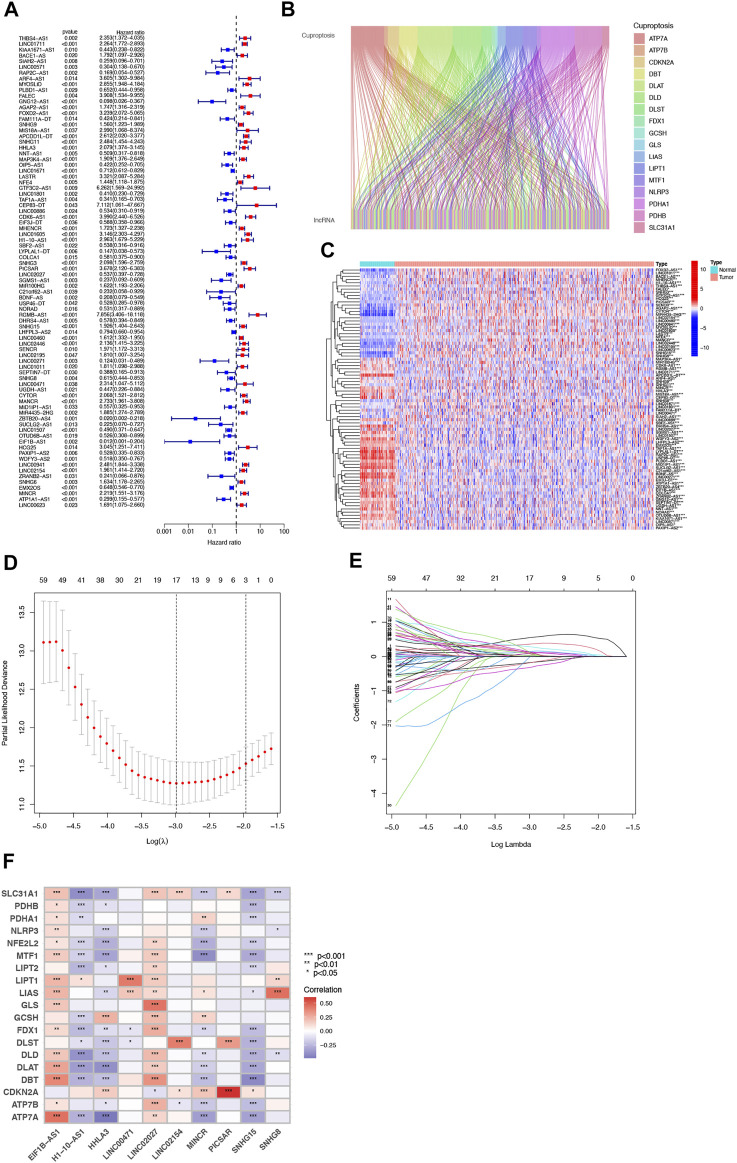
Identification of Cuproptosis-associated lncRNA prognostic features in ccRCC. The forest plot shows prognosis-related genes for cuproptosis-associated lncRNAs **(A)**. Sankey relationship diagram of cuproptosis genes and cuproptosis-associated lncRNAs **(B)**. Differential expression of 81 cuproptosis-associated lncRNAs associated with survival between ccRCC and normal samples **(C)**. Distribution of the LASSO coefficients of cuproptosis-associated lncRNAs **(D)**. The 10-fold cross-validation of variable selection in the least absolute shrinkage and selection operator (LASSO) algorithm **(E)**. Correlation of lncRNAs with cuproptosis-related genes in risk models **(F)**.

Our team identified 434 lncRNAs with co-expression relationships in ccRCC (|Pearson R| > 0.4 and *p* < 0.001) (Figure 1B). Univariate Cox analysis (*p* < 0.05) was utilized to choose 81 differentially expressed prognostic-related lncRNAs: THBS4-AS1, LINC01711, MACORIS, KIAA1671-AS1, BACE1-AS, SIAH2-AS1, LINC00571, RAP2C-AS1, ARF4-AS1, MYOSLID, PLBD1-AS1, FALEC, GNG12-AS1, AGAP2-AS1, OXCT1-AS1, FOXD2-AS1, SNHG9, LINC00882, APCDD1L-DT, SNHG11, OXCT1-AS1, CTBP1-DT, HHLA3, NNT-AS1, MAP3K4-AS1, OIP5-AS1, LINC01671, LASTR, NFE4, GTF3C2-AS1, LINC01801, LINC00886, CDK6-AS1, EIF3J-DT, MHENCR, LINC01605, H1-10-AS1, SBF2-AS1, PCCA-DT, LYPLAL1-DT, COLCA1, SNHG3, GAS6-DT, LINC02027, SGMS1-AS1, BDNF-AS, KLHL7-DT, NORAD, DHRS4-AS1, SNHG15, LHFPL3-AS2, LINC00460, LINC02446, LINC02195, LINC00271, GATA2-AS1, LINC01011, SEPTIN7-DT, SNHG8, UGDH-AS1, CYTOR, MANCR, MIR4435-2HG, ITGA9-AS1, ZBTB20-AS4, SUCLG2-AS1, LINC01507, OTUD6B-AS1, EIF1B- AS1, HCG25, PAXIP1-AS2, WDFY3-AS2, TGFB2-AS1, BAALC-AS1, LINC00941, LINC02154, SNHG6, EMS2OS, MINCR, ATP1A1-AS1, LINC00623, and LINC01415 (Figure 1A and C).

### Construction of the Cuproptosis-Related LncRNA Predictive Signature

**FIGURE 2:**
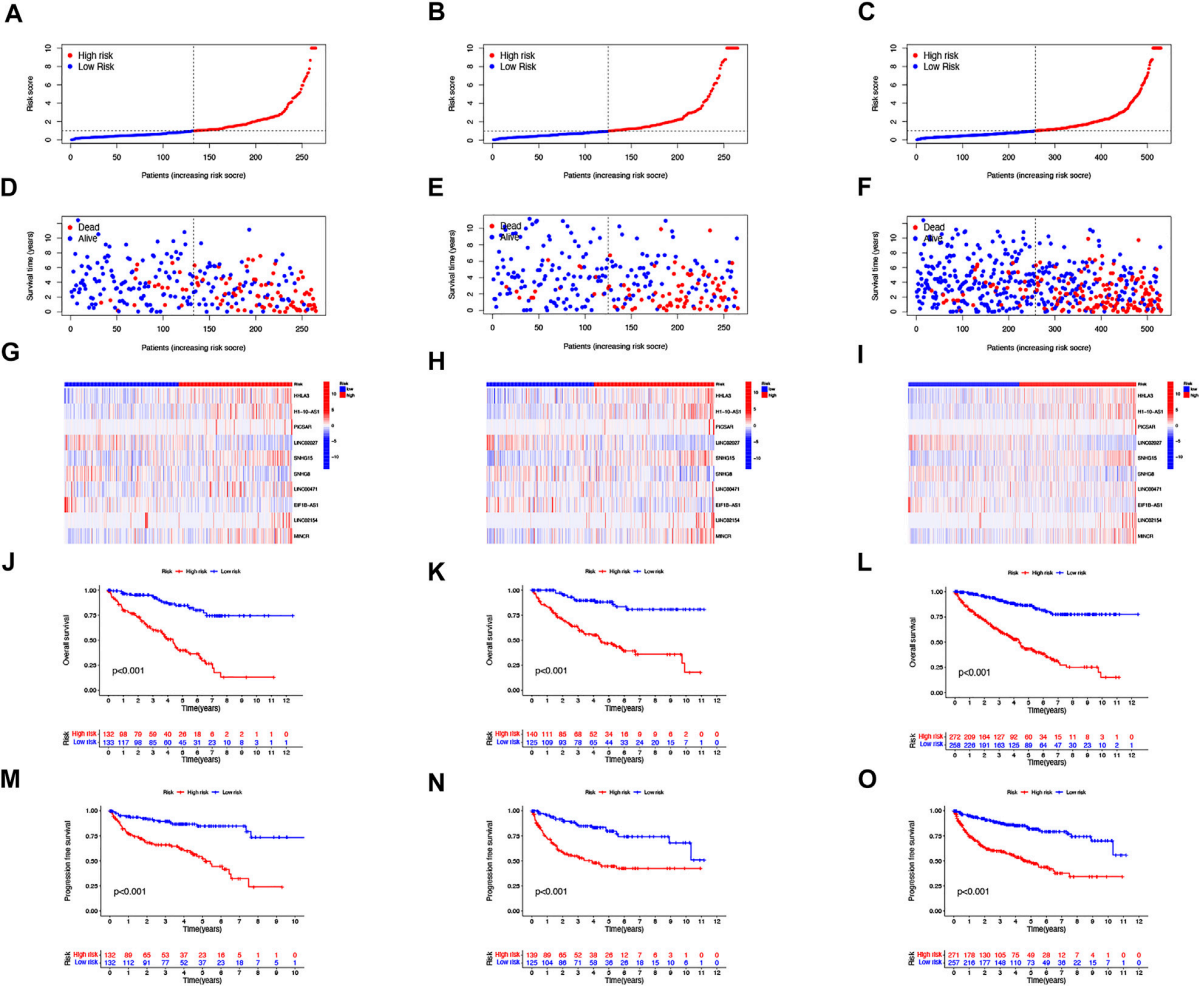
Prognosis of the risk model in different groups. The distribution of overall survival risk scores **(A–C)**, survival time and survival status **(D–F)**, heat maps of 10 lncRNA expressions **(G–I)**, Kaplan–Meier survival curves of overall survival of ccRCC patients **(J–L)**, and Kaplan–Meier survival curves of progression-free survival of ccRCC patients **(M–O)** between low- and high-risk groups in the train, test, and entire sets, respectively.

**FIGURE 3:**
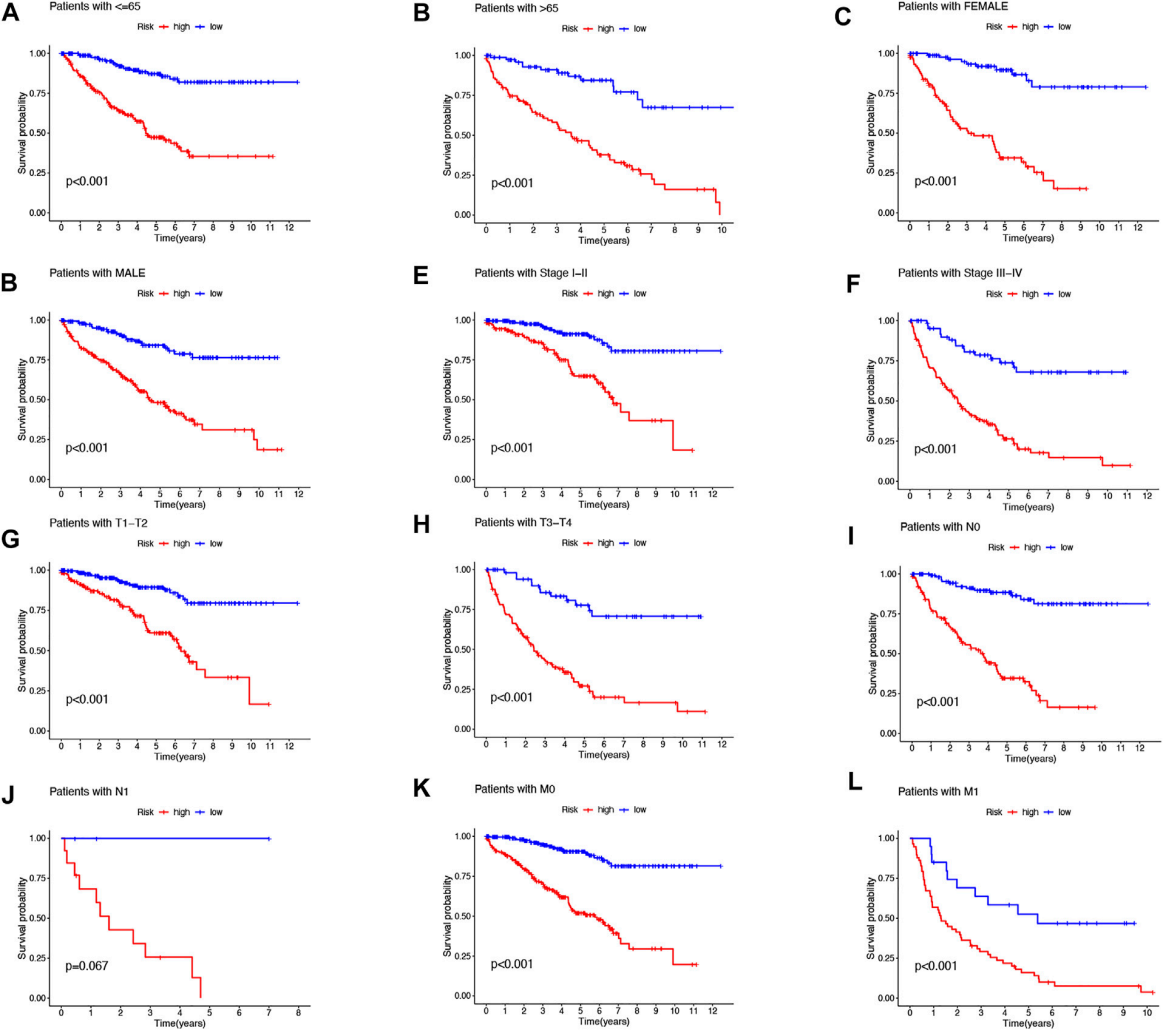
Kaplan–Meier survival curves for low- and high-risk populations by different clinical variables. Age **(A**,**B)**, sex **(C**,**D)**, stage **(E**,**F)**, T stage **(G**,**H)**, N stage **(I**,**J)**, and M stage **(K**,**L)**.

Then, we performed a LASSO Cox regression analysis using the training set and obtained the lncRNAs with the highest prognostic values using the “glmnet” package of R software (Figure 1D–F). Finally, we obtained 17 lncRNAs, 10 of which were introduced into the multi-Cox proportional risk model. The risk score was obtained using the multivariate Cox regression formula: risk score = HHLA3 × (0.4223) + H1-10-AS1 × (0.5960) + PICSAR × (0.9702) + LINC02027 × (−0.5392) + SNHG15 × (0.3602) + SNHG8 × (−0.6352) + LINC00471 × (1.2766) + EIF1B-AS1 × (−3.8776) + LINC02154 × (0.7232) + MINCR × (0.3724). Overall survival was significantly shorter for all patients in the high-risk group in the complete set and training and validation partitions (Figure 2A–L). Similarly, the progression-free survival was significantly lower in the high-risk group compared to the low-risk group (Figure 2M–O). Meanwhile, ccRCC patients were grouped by age, sex, stage, T-stage, N-stage, and M-stage to investigate the correlation between survival probability and risk score in generic clinicopathological characteristics. The results showed that for different classifications, except for stage N1 (Figure 3J), the overall survival rate was much higher in the low-risk group (Figures 3A–I, Figure 3K-L). A possible interpretation of the N1 stage was the limited number of patients because of the bad prognosis of advanced ccRCC. The results suggested that the model can be used to help predict the prognosis of patients with ccRCC with different clinicopathological variables.

### An Independent Prognostic Indicator of ccRCC of the Cuproptosis-Related lncRNA Signature

**FIGURE 4:**
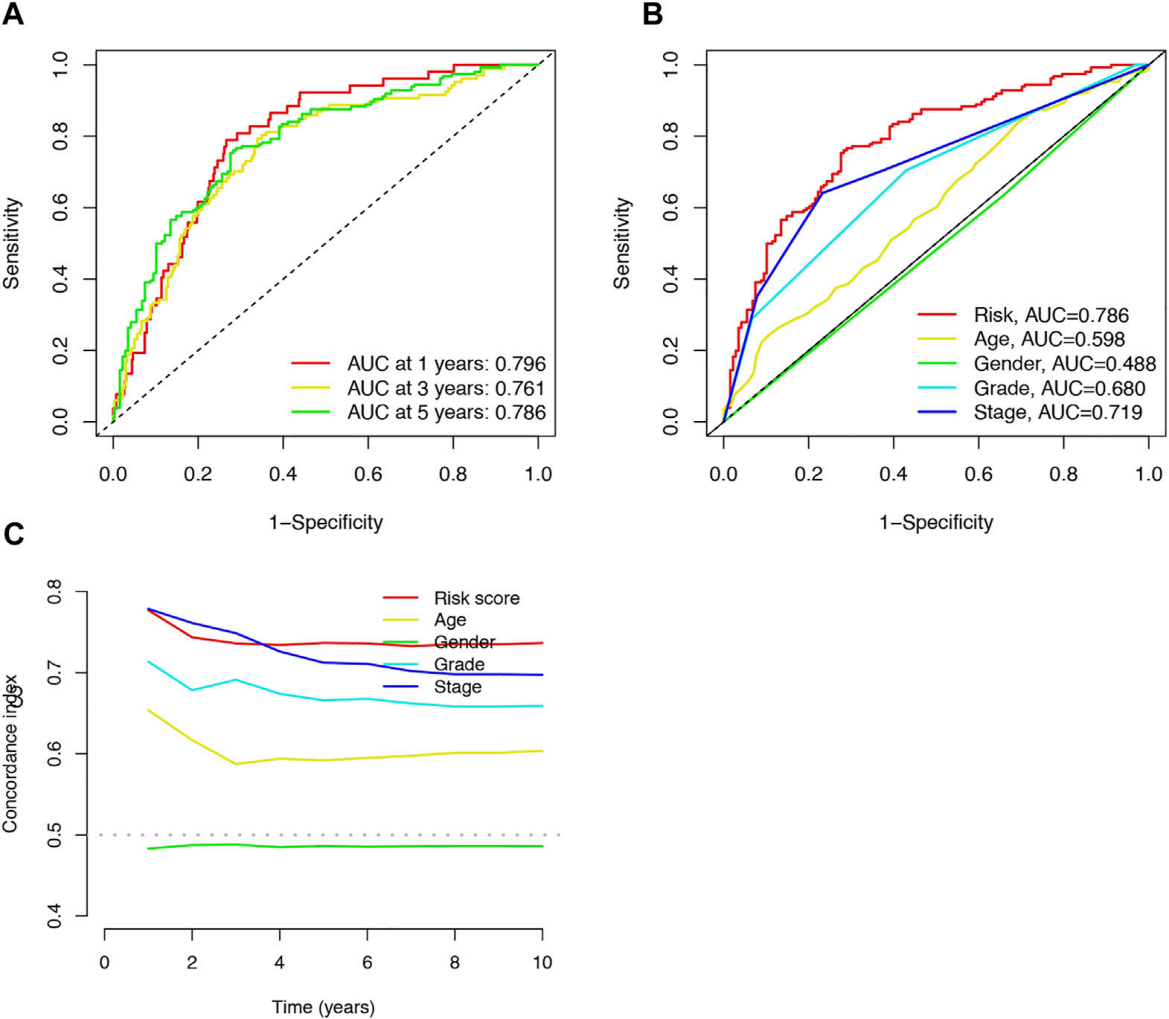
Accuracy of the risk characteristic based on a whole-group prediction of 1-, 3-, and 5-years receiver operating characteristic curves **(A)**. Predictive accuracy of the risk model compared with clinicopathologic characteristics such as age, sex, and stage **(B)**. C-index curve of the risk model **(C)**.

The area under the curve (AUC) was 0.796, 0.761, and 0.786 for the 1-, 3-, and 5-years ROCs, respectively (Figure 4A). The AUC of the risk score was 0.786 in the 5-years ROC of the model, showing extremely strong predictive power compared to other clinicopathological characteristics (Figure 4B). The 10-years C-index in the risk model was also higher than the other clinical features (Figure 4C).

### Construction and Validation of the lncRNA-Based Nomogram

**FIGURE 5:**
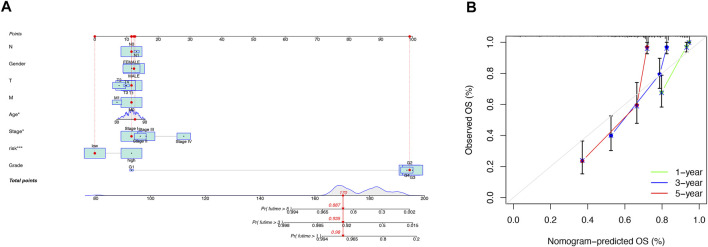
Construction and validation of the nomogram. A nomogram combining clinicopathological variables and risk scores predicts 1-, 3-, and 5-years overall survival in patients with ccRCC **(A)**. Calibration curves test the agreement between actual and predicted outcomes at 1, 3, and 5 years **(B)**.

Our team predicted the prognosis of ccRCC patients at 1, 3, and 5 years by constructing a nomogram that included clinical characteristics and risk scores (Figure 5A). The calibration curves showed good agreement between the nomogram and the predicted results (Figure 5B).

### The Principal Component Analysis and Biological Pathways Analyses

**FIGURE 6:**
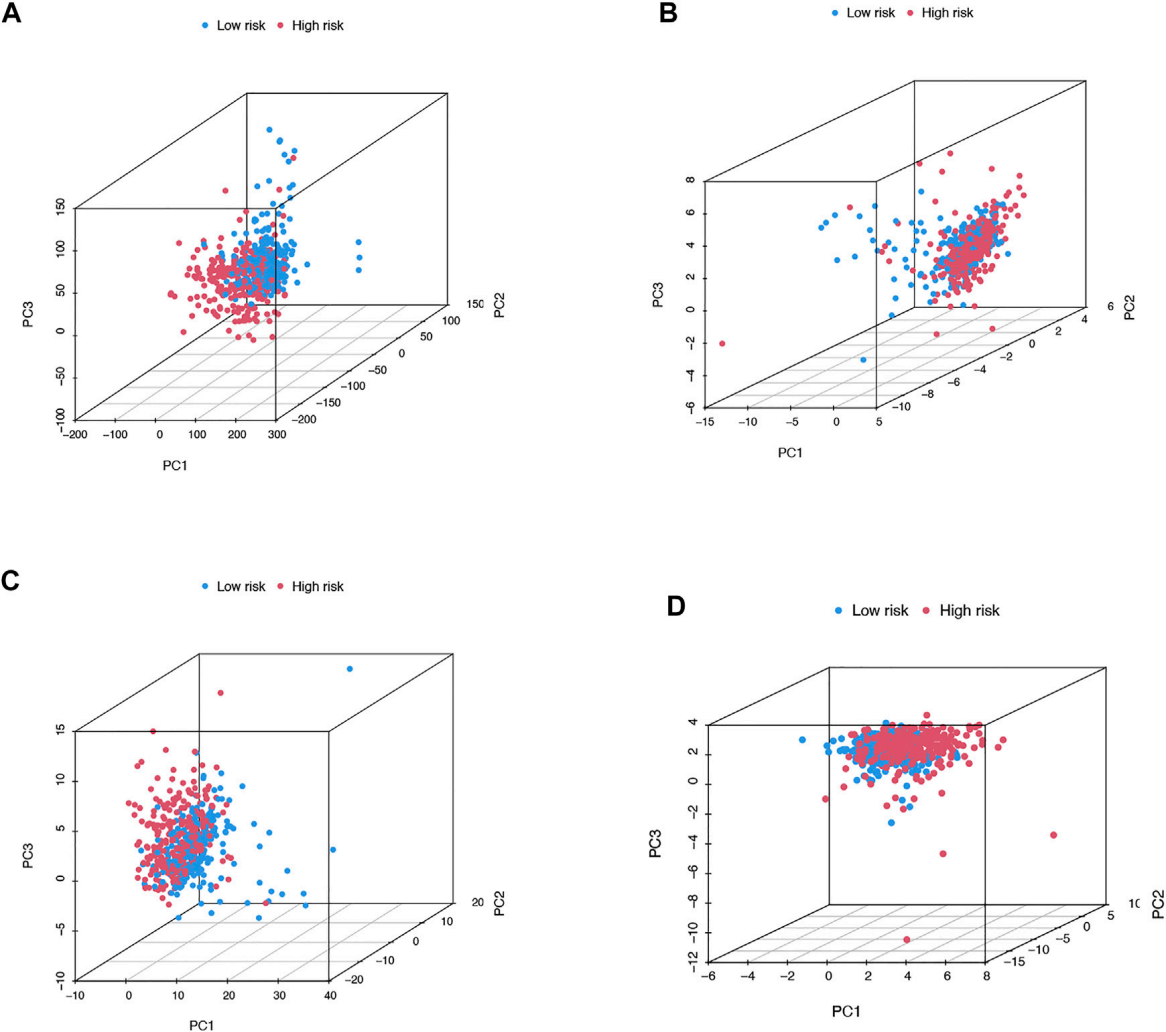
PCA in both groups of patients. PCA of all genes **(A)**. PCA of cuproptosis genes **(B)**. PCA of cuproptosis-related lncRNAs **(C)**. PCA of risk lncRNAs **(D)**.

**FIGURE 7:**
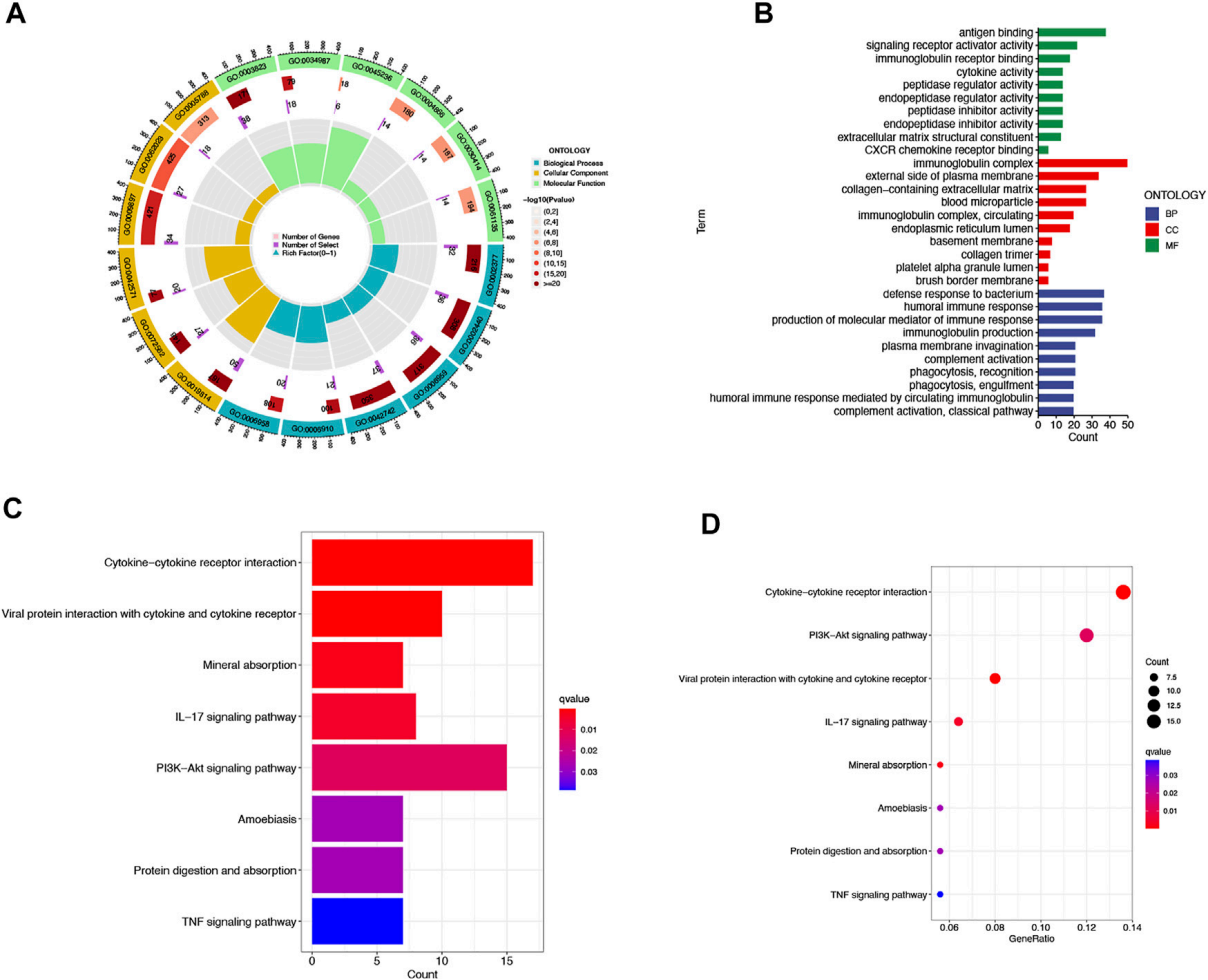
GO and KEGG analysis. Gene Ontology (GO) analysis demonstrated the richness of molecular biological processes (BP), cellular components (CC), and molecular functions (MF) **(A**,**B)**. KEGG pathway analysis showed the significantly enriched pathways **(C**,**D)**.

We then utilized PCA to explore the differences between the high- and low-risk groups in four expression profiles (total gene expression profiles, cuproptosis genes, cuproptosis-associated lncRNAs, and risk models classified by the expression profiles of 10 cuproptosis-associated lncRNAs) (Figure 6A–D). The outcomes indicated that the 10 cuproptosis-associated lncRNAs were of best discriminatory capacity to distinguish well between low- and high-risk populations. GO analysis showed that cuproptosis-associated lncRNAs were strongly associated with the development of immune responses (Figure 7A and B). KEGG analysis resulted mainly in cytokine–cytokine receptor interaction and PI3K-AKT signaling pathway (Figures 7C and D).

### Examination of Immune Characteristics in High- and Low-Risk Groups

**FIGURE 8:**
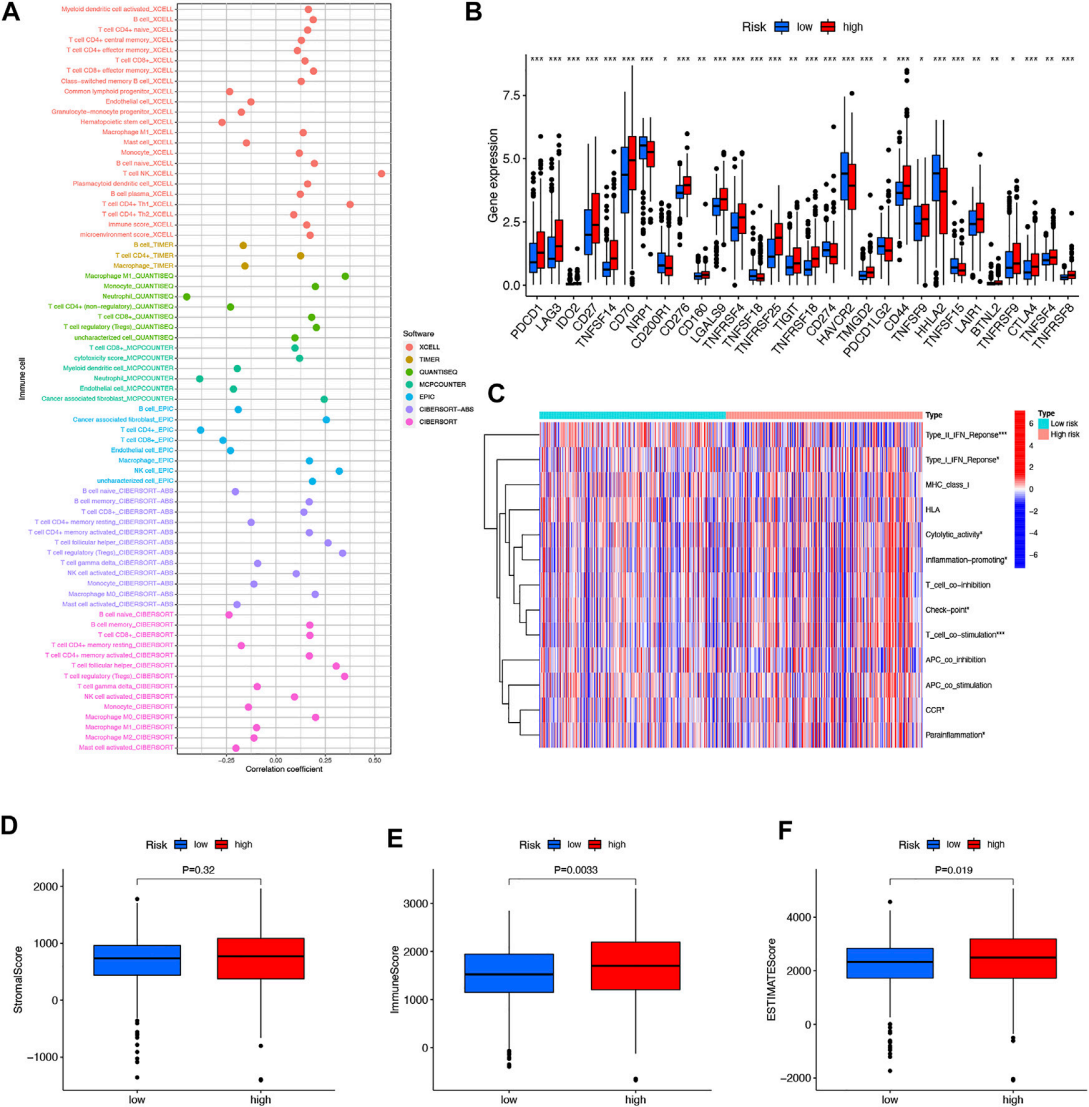
Differences in the tumor immune microenvironment between the low- and high-risk groups. Immune cell bubble of risk groups **(A)**. Differences in expression of common immune checkpoints in the risk groups **(B)**. ssGSEA scores of immune cells and immune function in the risk group **(C)**. Box plots comparing StromalScore, ImmuneScore and ESTIMATEScore between the low- and high-risk groups, respectively **(D–F)**. *p < 0.05, **p < 0.01, and ***p < 0.001.

In immune cell bubble graphs, our team found that samples from the high-risk group were significantly positively correlated with infiltration of regulatory T cells, B cell memory, NK cells, and T cell follicular helper and negatively correlated with neutrophil infiltration (all *p* < 0.05) (Figure 8A). Details of the infiltration of the aforementioned cells are shown in Supplementary Figure S1. In addition, we analyzed the differences in immune checkpoints between the high-risk and low-risk groups (Figure 8B). Interestingly, most of the immune checkpoints had higher expression in the high-risk patients, which may explain the poorer OS in the high-risk group. Subsequently, our team investigated the connection between risk scores and immune-related activities in ccRCC. The box plots of the results indicated that type II IFN response, Type I IFN response, cytolytic activity, inflammation-promoting, check point, T-cell co-stimulation, CCR, and parainflammation were dramatically different in the risk scores (Figure 8C). In terms of TME scores, immune scores and ESTIMATE scores were higher in high-risk patients than in low-risk patients, with no difference in stromal scores between them (Figure 8D–F).

### TMB, TIDE, and Therapeutic Drug Sensitivity

**FIGURE 9:**
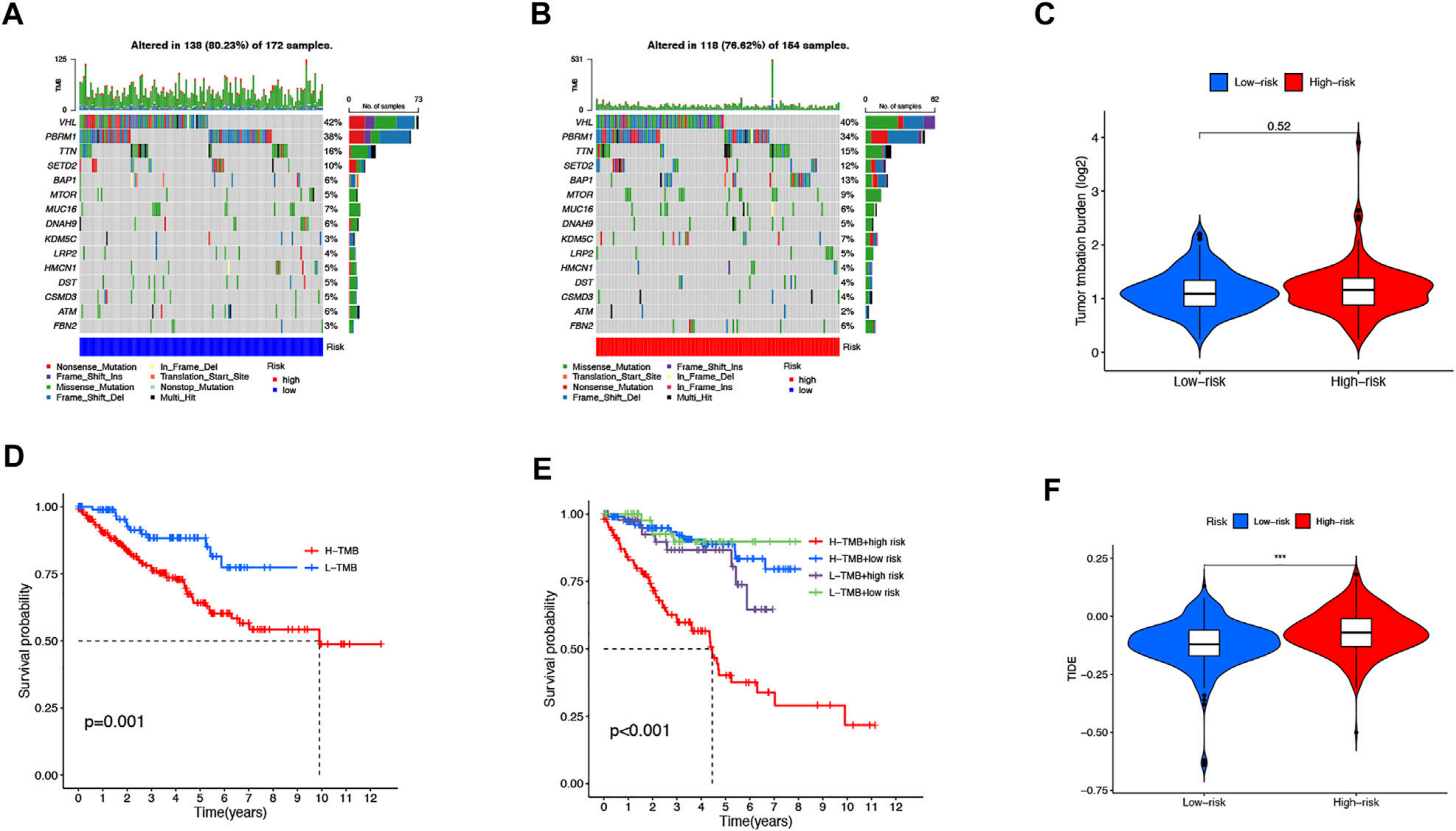
TMB, TIDE, and Chemotherapeutic Sensitivity. Waterfall plots of somatic mutation characteristics in the two groups **(A-B)**. TMB between the low-risk and high-risk groups **(C)**. K–M survival curves between the high- and low-TMB groups **(D)**. K–M survival curves between the four groups **(E)**. TIDE scores between the two groups **(F)**.

**FIGURE 10:**
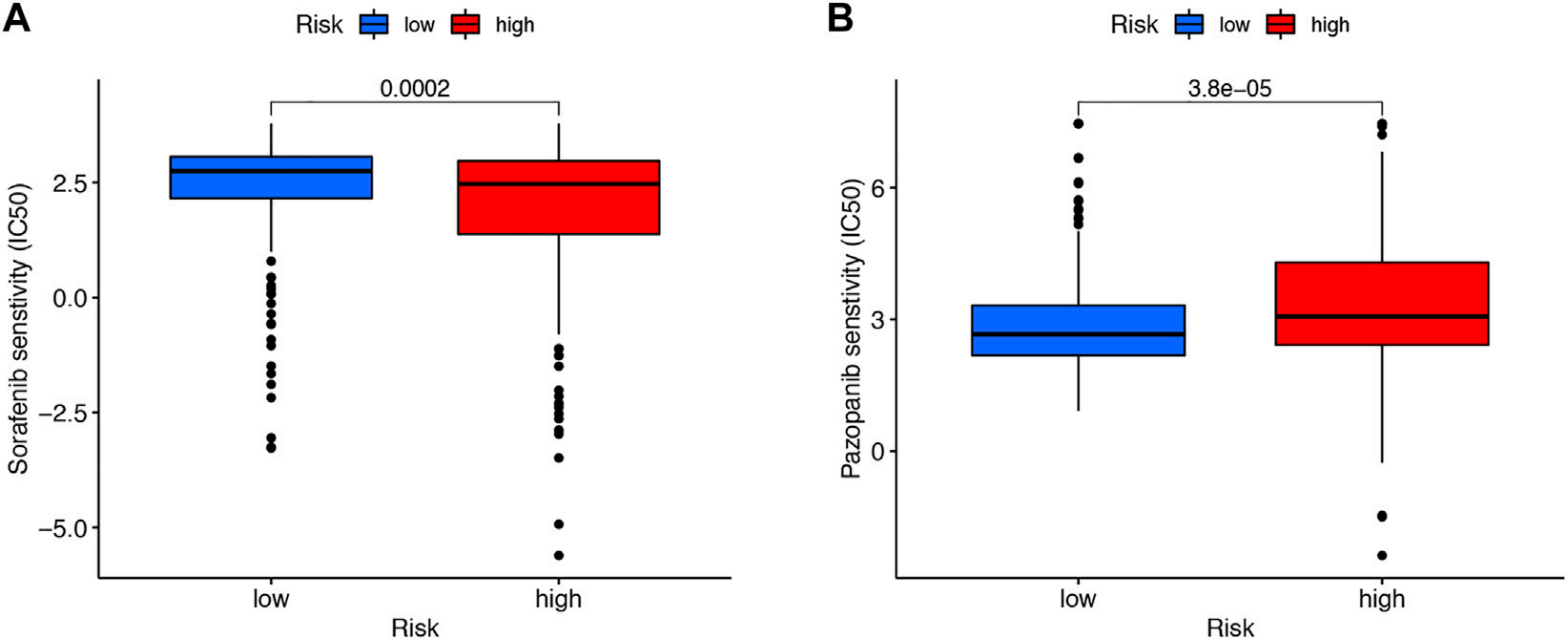
Drug sensitivity. Sorafenib was more effective in the high-risk group **(A)**. Pazopanib was more effective in the low-risk group **(B)**.

We then downloaded the somatic mutation data from the TGCA database and analyzed the changes in somatic mutations in the high- and low-risk groups. The 10 most highly mutated genes were VHL, PBRM1, TTN, SETD2, BAP1, MTOR, MUC16, DNAH9, KDM5C, and LRP2. (Figure 9A and B). Among these genes, VHL, PBRM1, SETD2, BAP1, KDM5C, and MTOR were the most frequently mutated genes in ccRCC. However, in general, there was no significant difference in TMB between the two groups (Figure 9C). In addition, patients in the high TMB and high-risk cohorts had the worst prognosis than the other groups (Figures 9D and E). Compared to the low-risk group, the TIDE scores were dramatically higher in the high-risk group (Figure 9F). By comparing drug sensitivity, we found significant differences in IC50 values between the low- and high-risk groups for multiple drugs. Drugs sensitive to the high-risk group and drugs sensitive to the low-risk group are shown in Supplementary Figures S2 and S3, respectively. Of these drugs, sorafenib was more effective in the high-risk group and, conversely, pazopanib was more effective in the low-risk group (Figure 10A and B).

### External Validation of Cuproptosis-Related lncRNAs as a Potential Biomarker

**FIGURE 11:**
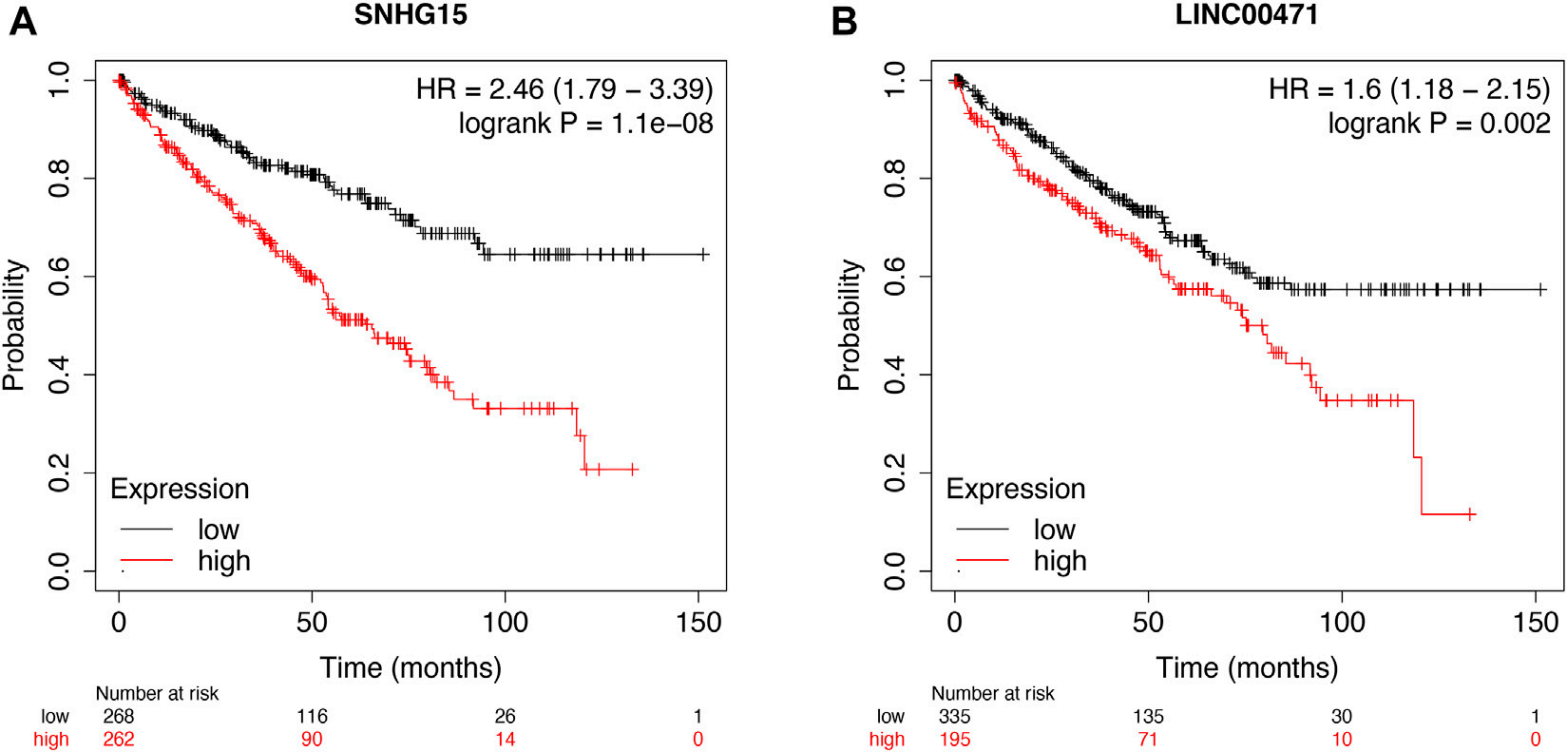
External validation of cuproptosis-associated lncRNAs as potential biomarkers. OS analysis of SNHG15 and LINC00471 in the Kaplan–Meier Plotter datasets **(A** and **B)**.

Then, the KM survival analysis was utilized to verify the prognostic value of SNHG15 and LINC00471 in the external Kaplan–Meier Plotter database. The results showed that SNHG15, as a poor prognostic factor, was dramatically correlated with OS (HR = 2.46 (1.79–3.39), Log-rank *p* = 1.1e-08) (Figure 11A). LINC00471, an indicator of bad prognosis, was also significantly associated with OS (HR = 1.6 (1.18–2.15), Log-rank *p* = 0.002) (Figure 11B). The results of the survival analysis of external datasets were consistent with our outcomes.

### *In Vitro* Experimental Validation of Cuproptosis-Related lncRNAs as a Potential Biomarker

**FIGURE 12:**
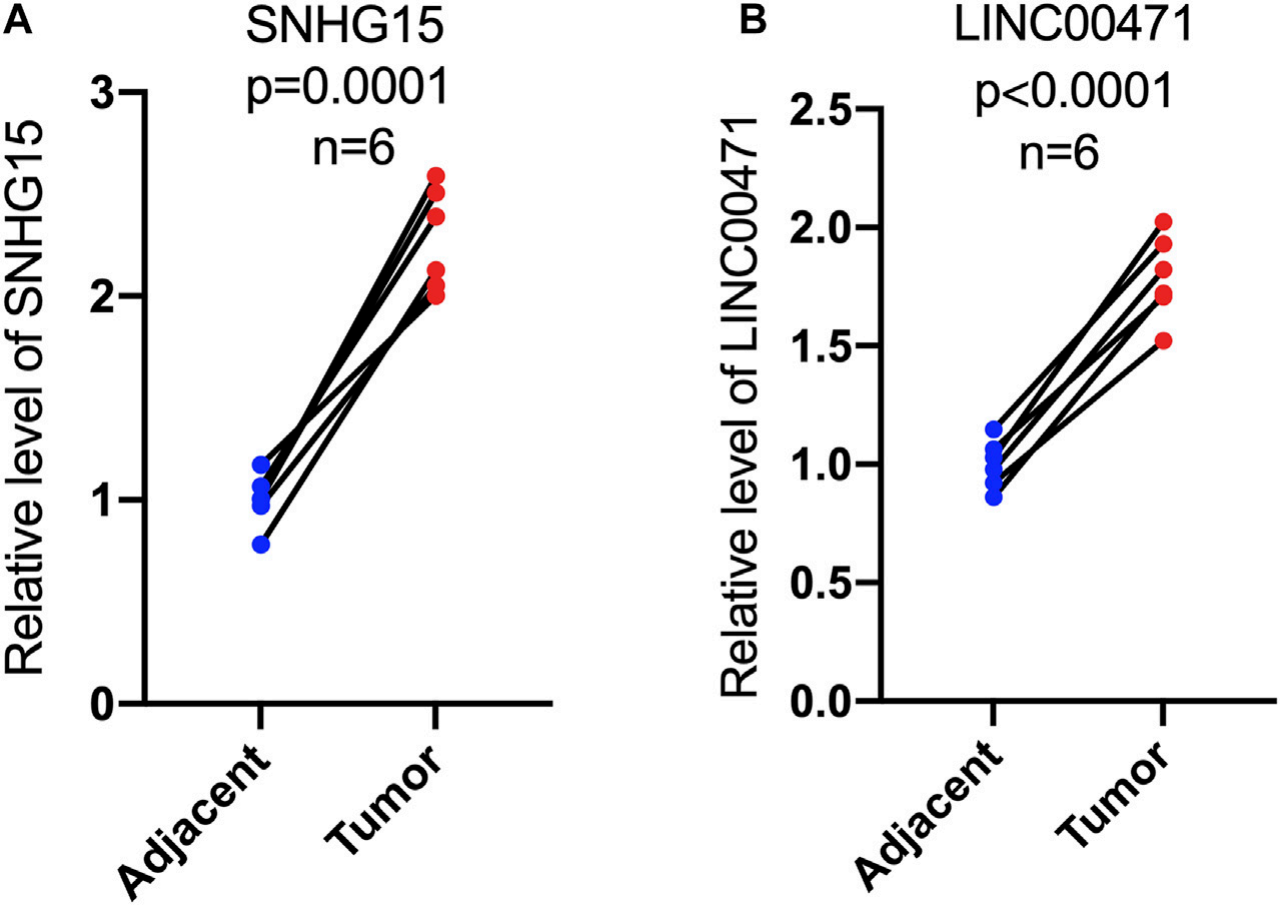
Expression levels of cuproptosis-associated lncRNAs in paired tumor tissues. RT-qPCR was used to measure the expression of SNHG15 and LINC00471 in paired tumor tissues **(A**,**B)**.

To further validate the prognostic value of this cuproptosis death-associated lncRNA model, our team performed *in vitro* experiments to illustrate the expression trends of hub differentially expressed cuproptosis-associated lncRNAs. RT-qPCR results indicated an overall trend of increased SNHG15 and LINC00471 expression levels in ccRCC tissues compared to adjacent paired normal tissues, which matched the results of our previous bioinformatics analysis based on public databases (Figure 12A and B).

## Discussion

CCRCC, as the most aggressive subtype, is the predominant histological type of renal cancer.

Although surgical resection is a moderate treatment option for localized ccRCC, the outcome of advanced or metastatic ccRCC remains dissatisfactory. Therefore, the identification of prospective prognostic and molecular signatures specific to patients with ccRCC is essential to improve the patient’s prognosis.

Recent studies have shown that intracellular copper accumulation triggers the aggregation of mitochondrial lipid acylated proteins and the loss of Fe–S cluster proteins, resulting in a proteotoxic stress-induced death called cuproptosis (Tsvetkov et al., 2022). Significantly, the accumulation of intracellular copper is dependent on the transport of copper ionophores. Therefore, copper ionophores are a powerful tool for studying copper toxicity (Hunsaker and Franz, 2019). Traditional cancer treatments usually harm normal cells, so novel therapeutic agents are being developed with the aim of improving selectivity and, thus, reducing side effects. In addition, these agents should target cancer stem cells, thus, overcoming the resistance of cancer cells. Cancer cells are usually preferentially induced by cuproptosis compared to normal cells, and some copper ionophores have shown promise in this direction (Li, 2020; Steinbrueck et al., 2020; Babak and Ahn, 2021; Michniewicz et al., 2021; Shanbhag et al., 2021). Therefore, cuproptosis-related studies are urgently needed for a deeper understanding.

Previous studies have shown that lncRNAs play an important regulatory role in the development and progression of ccRCC. Professor Liu confirmed that LINC01232 promotes clear cell renal cell carcinoma by binding miR-204-5p to upregulate RAB22A (Liu et al., 2021). Lv noted that the long non-coding RNA TUG1 promotes cell proliferation through the MIR-31-5p/FLOT1 axis in clear cell renal cell carcinoma and inhibits apoptosis and autophagy (Lv et al., 2020). However, lncRNAs associated with cuproptosis have never been studied in ccRCC. Here, our team constructed a cuproptosis-associated lncRNA signature to predict the prognostic status of ccRCC patients. In our research, we obtained 81 cuproptosis-related lncRNAs associated with prognosis by analysis. We screened and identified 10 cuproptosis-related lncRNAs significantly associated with OS by univariate, LASSO, and multivariate Cox regression analysis (HHLA3, H1-10-AS1, PICSAR, LINC02027, SNHG15, SNHG8, LINC00471, EIF1B-AS1, LINC02154, and MINCR). With the aforementioned lncRNAs, we constructed cuproptosis-related lncRNA features to predict the prognosis of ccRCC patients. Among these lncRNAs, the lncRNA PICSAR was reported to be highly expressed in tumors and could promote proliferation and migration and inhibit apoptosis in cutaneous squamous cell carcinoma and hepatocellular carcinoma (Liu et al., 2020; Lu et al., 2021). LINC02027 was an important member of the ccRCC prognostic model (Chen et al., 2022). LncRNA SNHG15 was a novel lncRNA identified as a tumor promoter in various human cancers, including hepatocellular carcinoma (HCC), colorectal cancer (CRC), breast cancer (BRCA), pancreatic cancer (PC), gastric cancer (GC), and clear cell carcinoma (ccRCC) (Guo et al., 2018; Jin et al., 2018; Kong and Qiu, 2018; Huang et al., 2019; Yang et al., 2020; Chen et al., 2021). Studies in ccRCC have shown that increased expression of lncRNA SNHG15 was an independent predictor of shorter RFS. In addition, SNHG15 expression levels were significantly regulated by DNA methylation in ccRCC (Yang et al., 2020). All findings suggested that SNHG15 was promising as a biomarker and therapeutic target for cancer patients. Similarly, SNHG8 was considered to be an oncogenic factor and was upregulated in various types of cancer (Yuan et al., 2021), such as gastric cancer, melanoma, nasopharyngeal cancer, and esophageal cancer (Shan et al., 2022b; Luan et al., 2022; Wu et al., 2022; Zhu et al., 2022). LINC00471 was an essential member of the prognostic model of childhood acute myeloid leukemia and esophageal squamous cell carcinoma (Zhang et al., 2019a; Yu et al., 2019). LINC02154 was involved in the construction of a prognostic model for laryngeal squamous cell carcinoma (Zhang et al., 2019b; Gong et al., 2020). MINCR was highly expressed in nasopharyngeal, colon, non-small cell lung cancers, and hepatocellular carcinoma and promotes cancer development (Cao et al., 2018; Chen et al., 2019; Yu et al., 2020; Zhong et al., 2020). The remaining three lncRNAs (HHLA3, H1-10-AS1, and EIF1B-AS1) are the first publicly available. In particular, these newly discovered cuproptosis-related lncRNAs can help us better understand ccRCC and find new targets for cancer therapy. We then divided patients with ccRCC into low-risk and high-risk cohorts according to median values. The Roc and c-index curves were used to validate the prognostic accuracy of the risk score. We could find that the risk score could be used as a criterion to predict the prognosis. Then, we constructed a nomogram to predict the prognosis of patients with ccRCC. The calibration curves showed excellent agreement between actual results and predictions. Then the PCA results showed that the 10 cuproptosis-associated lncRNAs had the best ability to discriminate well between low- and high-risk populations. GO analysis suggested that immune responses were strongly associated with lncRNAs associated with cuproptosis. KEGG analysis showed that cytokine–cytokine receptor interactions and the PI3K-AKT signaling pathway were most active in cuproptosis-associated lncRNAs. The PI3K-Akt signaling pathway was widely present in a variety of cells and can be involved in cell proliferation, apoptosis, invasion, metastasis, and angiogenesis by altering the activation status of downstream signaling molecules, which had been regarded by scientists as the primary pathway for cancer cell survival (Polivka and Janku, 2014). Normally, immune cell infiltration in the tumor microenvironment varies with tumor progression. Sierra et al. (2021) found *in vitro* experiments that an increase in NK cells suppressed the proliferation of CD8^+^ T cells and suggested that infiltration of NK cells impairs the immune regulatory function of the body. A study showed that T cell follicular helper cells, T cell regulation, and B cell memory were associated with adverse outcomes of ccRCC (Yu et al., 2020). The characteristics of the high-risk group we established were highly consistent with the aforementioned study and predicted a poorer prognosis for the high-risk group. Furthermore, the results of ssGSEA pointed to an immune profile of type II inactivation of the IFN response and activation of T cell co-stimulation in high-risk populations. These results suggested that our features may be involved in the tumor immune microenvironment of ccRCC, acting by blocking the immune response, and may be a factor in the progression of ccRCC. We also performed immune scores, stromal scores, and ESTIMATE scores on different subgroups of the population, resulting in higher-risk groups having higher immune scores and lower tumor purity. As previously reported, the TIDE algorithm was used to assess the clinical response of patients to ICI therapy; the higher the TIDE score, the greater the likelihood of immune escape, which may imply a limited response and shorter survival time for patients treated with ICI. Compared to the low-risk group, patients in the high-risk group had higher TIDE scores, suggesting that patients in the high-risk group may have a more limited response to ICI therapy. Previous clinical trials have confirmed that the benefits of pazopanib are more prominent in the low-risk group, which is consistent with our study (Méndez-Vidal et al., 2018). However, in the case of sorafenib, there is no evidence in the literature that it is more beneficial in the high-risk group, and the exact mechanism remains to be confirmed by more studies. Our team constructed 10 copper death-associated lncRNAs to predict the prognosis of patients with ccRCC through adequate bioinformatics analysis. However, our study still had some drawbacks and shortcomings. First, we could not get validation from the GEO and ICGC databases. Even though we tried the GEO and ICGC databases, we still could not obtain proper lncRNA information due to the bias and limitation of commercial microarray data compared with GTEx and TCGA. Therefore, we validated the potential ability of two of these lncRNAs as biomarkers by PCR together with the external database Kaplan–Meier Plotter database. In addition, the immune cell bubble plots showed the results of immune infiltration from multiple platforms, which in a sense can be considered as external validation. In addition, our team will subsequently collect additional clinical datasets to validate the value of cuproptosis-associated lncRNAs.

## Conclusion

The 10 cuproptosis-related-associated lncRNA risk profiles may help to assess the prognosis and molecular profile of ccRCC patients and improve treatment options, which may be further applied in the clinic.

## Data Availability

The datasets presented in this study can be found in online repositories. The names of the repository/repositories and accession number(s) can be found in the article/Supplementary Material.
