# Supplementary material for: Cuproptosis-Associated lncRNA Establishes New Prognostic Profile and Predicts Immunotherapy Response in Clear Cell Renal Cell Carcinoma
Source: Front Genet. 2022 Jul 15;13:938259. doi: 10.3389/fgene.2022.938259 (PMC9334800; doi:10.3389/fgene.2022.938259)
Supplement: Supplementary file 1 [file DataSheet1.docx]

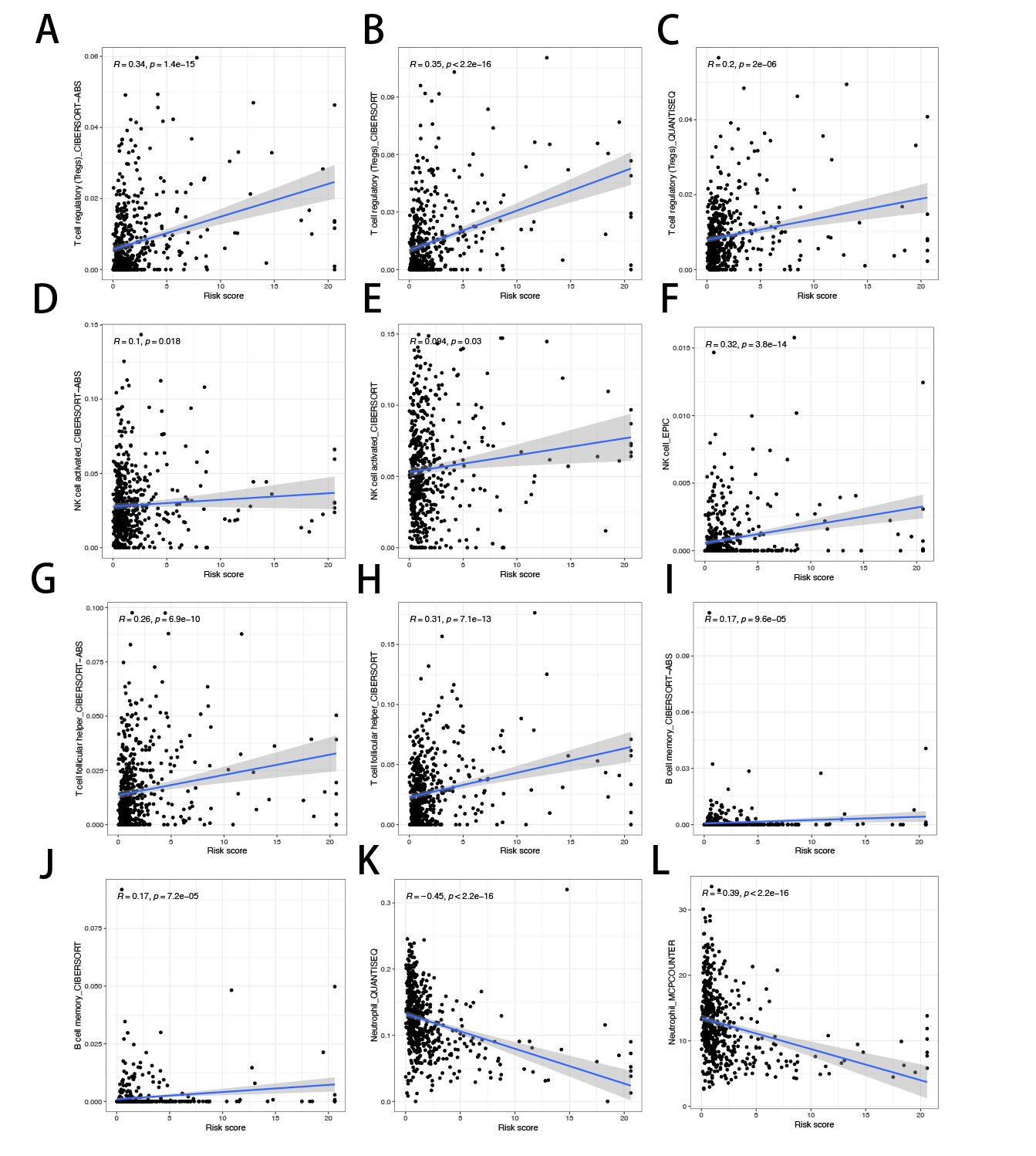


**Figure Supplementary Figure 1.** Correlation analysis between the level of immune cell infiltration and risk score. Regulatory T cells **(A-C)**, NK cells **(D-F)**, T cell follicular helpers **(G-H)**, B cell memory **(I-J)**, and immune infiltration of neutrophils **(K-L)** in relation to immune scores.


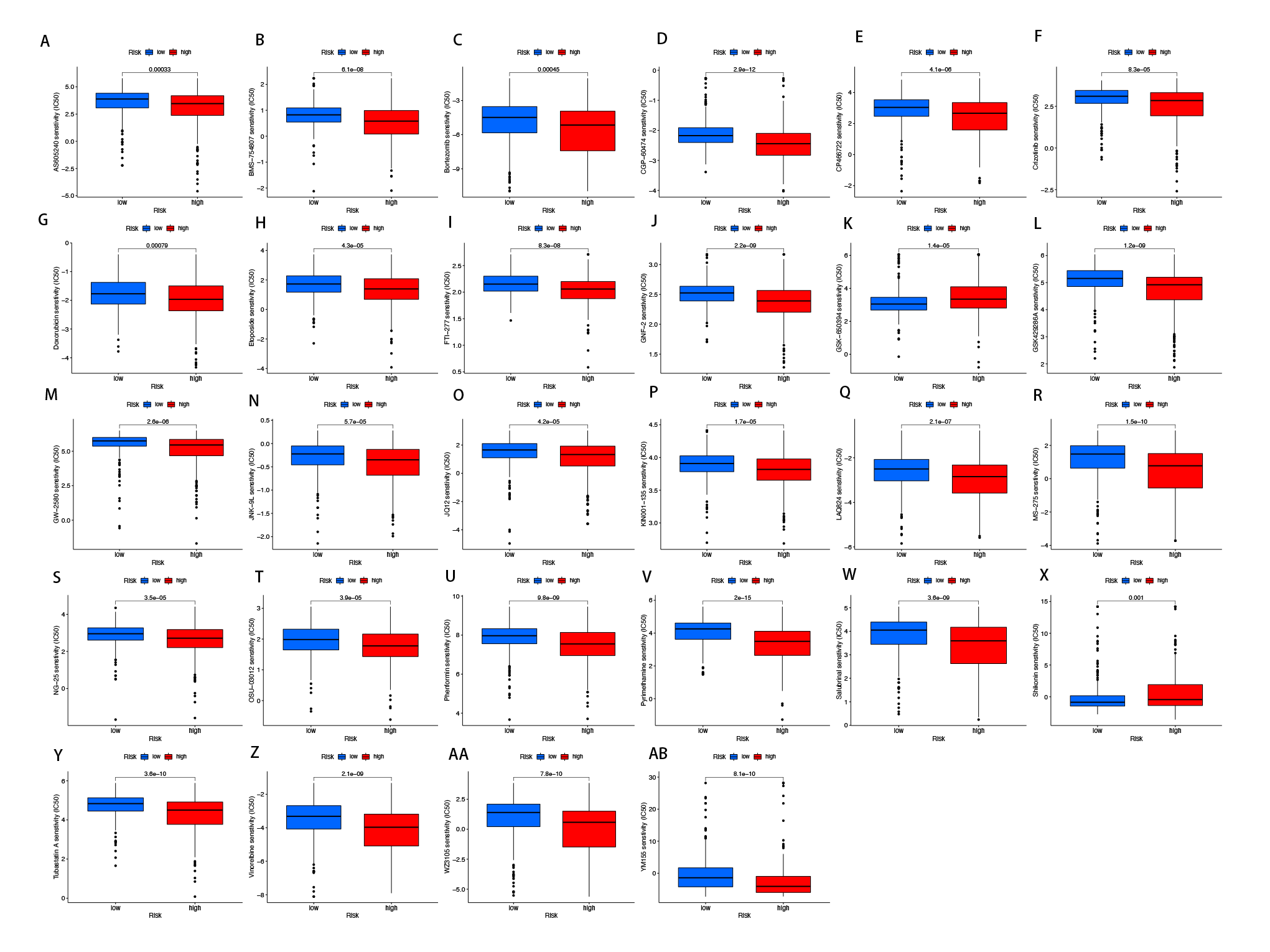


**Figure Supplementary Figure 2.** Drugs sensitive to high risk groups **(A-AB)**.


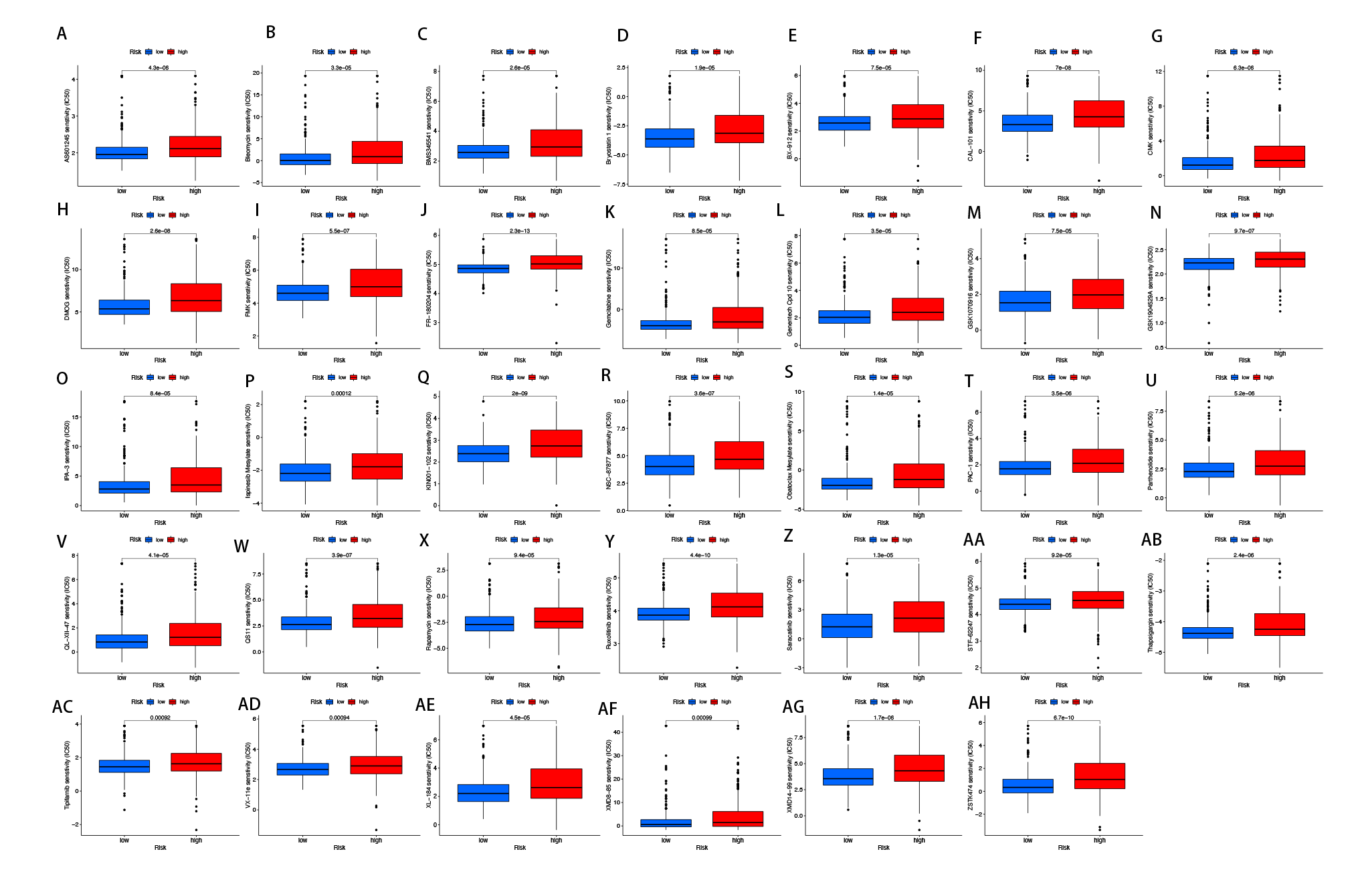


**Figure Supplementary Figure 3.** Drugs sensitive to low risk groups **(A-AH)**.
